# IsiA Is Required for the Formation of Photosystem I Supercomplexes and for Efficient State Transition in *Synechocystis* PCC 6803

**DOI:** 10.1371/journal.pone.0010432

**Published:** 2010-05-03

**Authors:** Qiang Wang, Camille L. Hall, Mustafa Z. Al-Adami, Qingfang He

**Affiliations:** Department of Applied Science, University of Arkansas, Little Rock, Arkansas, United States of America; Auburn University, United States of America

## Abstract

Iron deficiency and other stress conditions strongly impact photosynthetic apparatus in photosynthetic organisms. Two novel chlorophyll (Chl)-containing supercomplexes (F4 and F5) in addition to the photosystem (PS) I trimers (F3) were observed by sucrose gradient ultracentrifugation in *Synechocystis* PCC 6803 under extensive iron starvation. 77K fluorescence and Western blot analyses of these supercomplexes revealed that they all contained IsiA. The F4 was identified as an IsiA-PSI-PSII supercomplex, while the F5 was assigned as an IsiA-PSI supercomplex. Deletion of *isiA* resulted in diminishing the PSI trimers (including the PSI trimers in iron-replete cells) and the two novel PSI supercomplexes (F4 and F5), and a significant reduction in the saturated whole-chain electron transport rate. However, the maximum PSII activities remained at levels similar to those of the wild type under various light conditions. The *isiA^-^* mutant was defective in state transition and sensitive to high light. The sensitivity of the *isiA^-^* mutant to high light was correlated with a higher level of membrane peroxidation. These results demonstrated that IsiA is required for the formation of PSI trimers and other higher complexes, and that IsiA is critical for efficient state transition.

## Introduction

The photosynthetic apparatus of cyanobacteria, containing 22–23 iron atoms [1], is highly vulnerable to iron deficiency [1], [2]. Iron starvation results in various structural and functional changes within the cyanobacterial cells [3], [4], [5], [6], [7], [8], [9]; among these changes is the appearance of a chlorophyll (Chl)-protein complex associated with the *isiA* gene product IsiA, or CP43' [10], [11], [12], [13]. While the protein is most strongly induced by iron starvation and becomes the major Chl-binding protein within the cells under this condition [10], [14], [15], it has been reported to be inducible to some extent (at least transcriptionally) by other environmental stresses, including salt stress, heat stress, oxidative stress, and high or limited light conditions [16], [17], [18], [19], [20], [21], [22], [23]. These studies, however, did not provide evidence that *isiA* gene transcription under non-iron-limiting conditions is indeed accompanied by protein synthesis and membrane integration of IsiA [24]. It has been recently shown that the IsiA protein is enriched in the HLIP-containing PSI trimers prepared from cells treated with high-intensity light (HL) [25].

IsiA is widely distributed among cyanobateria, however, no homologs of IsiA were found in higher plants. The IsiA protein induced by iron starvation is known to encircle the PSI reaction centers, forming a protein supercomplex consisting of a trimeric PSI and a circle of 18 IsiA subunits [26], [27]. The oligomeric complexes of IsiA around PSI may increase the absorptional cross section of PSI and serve as a light-harvesting complex for the PSI complexes during iron starvation [28], [29]. IsiA can also form larger aggregates around the PSI monomer or in the total absence of PSI under longer term iron starvation conditions [30], [31]. These IsiA aggregates are in a strongly quenched state and they might be responsible for the thermal dissipation of absorbed energy [32], [33], [34]. It has been shown that IsiA is important for the survival of cyanobacterial cells in HL [18], [33], [34], [35]. The protein was also proposed to be essential for the blue light induced reversible non-photochemical fluorescence quenching under iron starvation conditions [33], [34], [36], [37]. However, Wilson et al. reported that the deletion of the IsiA does not affect the blue light-induced and the orange carotenoid protein (OCP)-related quenching processes under HL conditions [38], [39]. No biochemical analyses of IsiA or its associated protein complexes were performed in these studies.

In this study, we analyzed the Chl-containing protein complexes under extensive iron starvation conditions in *Synechocystis* PCC 6803. We observed for the first time the formation of a novel IsiA-PSI-PSII supercomplex and an IsiA-PSI supercomplex with higher density than that of the IsiA-PSI trimers by sucrose gradient ultracentrifugation. Deletion of *isiA* resulted in the diminishment of these supercomplexes and a significant reduction in the whole-chain electron transport rate. The i*siA^-^* mutant was defective in state transition. The sensitivity of the mutant to HL correlates with a higher level of membrane peroxidation. These results reveal the critical role of IsiA in the formation of photosynthetic supercomplexes and HL acclimation.

## Results

### IsiA-PSI complexes under extended iron stress conditions

**Figure 1:**
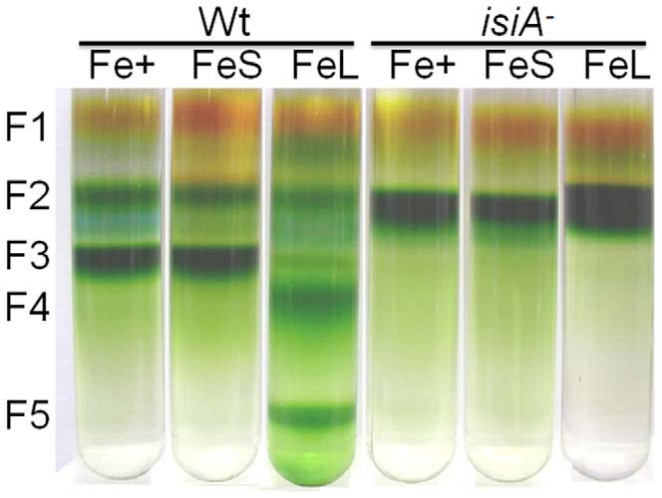
Fractionation of the thylakoid protein complexes by sucrose gradient ultracentrifugation. Wild type (Wt) and *isiA^-^* cells in mid-logarithmic growth (OD_730_ ∼0.6–0.8) were collected, washed and resuspended to an OD_730_ of 0.2 in either regular BG-11 (Fe+, control), or iron-free BG-11medium (iron depletion). Cells were further grown for 3 d (short-term iron depletion, FeS) or 10 d (long-term or extensive iron depletion, FeL). Thylakoid membranes were isolated and solubilized with mild detergent (dodecyl maltoside, with a 15∶1 detergent to Chl ratio). The thylakoid protein complexes were then separated by step sucrose gradient ultracentrifugation at 160,000×*g* for 16 h at 4°C.

The photosynthetic apparatus, especially PSI of cyanobacteria, is highly vulnerable to iron deficiency [1], [2]. To study how photosynthetic complexes react to iron stress, *Synechocystis* PCC 6803 cells grown in BG-11 medium at mid-logarithmic growth phase (OD_730_ approximately 0.6–0.8) were collected, resuspended with fresh iron-depletion medium [40] to an OD_730_ of 0.2, and further treated for 3 (short term, final OD_730_ ∼0.8) or 10 days (extended or long term iron stress, final OD_730_ ∼1.2). The thylakoid protein complexes were then isolated, solubilized in mild detergents and fractionated by sucrose gradient ultracentrifugation. The results are shown in Fig. 1. When cells were grown in iron replete medium in low light, the wild type samples contained typically three pigmented fractions: the free pigment fraction (F1), the PSI and PSII monomer fraction (F2), and the trimeric PSI fraction (F3). In contrast, the *isiA* ^-^ samples contained only the fractions from F1 and F2, and were completely lacking F3. Therefore, the integrity of the PSI trimers is severely affected by the deletion of *isiA*. This is a rather striking result as both *isiA* transcripts (analyzed by RT-PCR, data not shown) and the IsiA proteins (analyzed by Western Blot) are at very low levels under iron replete and low light (LL) conditions (please also see ref. [23]).

Under short-term iron stress conditions, the wild type cells maintained all three fractions. However, when the treatment was extended to 10 days, two additional heavier fractions of pigmented protein complexes, F4 and F5, were observed in the wild type samples, but not in the *isiA* ^-^ samples (Fig. 1).

**Figure 2:**
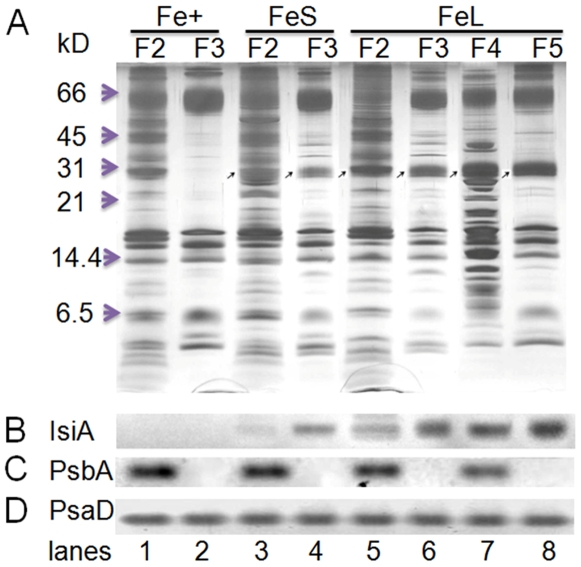
SDS-PAGE and Western blot analyses of the sucrose-gradient fractions. Green fractions from Fig. 1 were collected, denatured, and separated (30 µg proteins/lane) by SDS-PAGE (A). The proteins were blotted onto nitrocellulose membranes and probed with anti-IsiA (B), anti-PsbA (C) and anti-PsaD (D) antibodies. The IsiA protein bands in A were indicated by thin Arrows.

To investigate whether IsiA was incorporated into any of the pigmented fractions, we performed Tricine SDS-PAGE and Western blot analyses on the fractions. Fig. 2A showed that IsiA was present in both short-term (3 days) and long-term (10 days) iron-stressed fractions (indicated by arrowheads in the figure), which was confirmed by the Western blots with anti-IsiA antibodies (Fig. 2B). As compared to the iron-stressed samples (Fig. 2A, lanes 4 and 6), the F3 fraction (Fig. 2A, lane 2) of the control sample (cells grown in normal BG 11 medium in LL) lacked the IsiA protein. Interestingly, the protein profiles of F4 (a fraction with higher density, Fig. 2A, lane 7) were similar to those of F2 (Fig. 2A, lanes 3 and 5); they were both a mixture of PSI and PSII. The protein profiles of F5 (Fig. 2A, lane 8) were similar to F3, which mainly consisted of PSI proteins. Western blot analyses with anti-PsbA (Fig. 2C) and anti-PsaD (Fig. 2D) antibodies confirmed that the F5 (Fig. 2, lane 8) and all F3 fractions (Fig. 2, lanes 2, 4 and 6) are PSI containing but free of PSII, while the F4 (Fig. 2, lane 7) and all F2 (Fig. 2, lanes 1, 3 and 5) fractions are mixtures of PSI and PSII.

**Figure 3:**
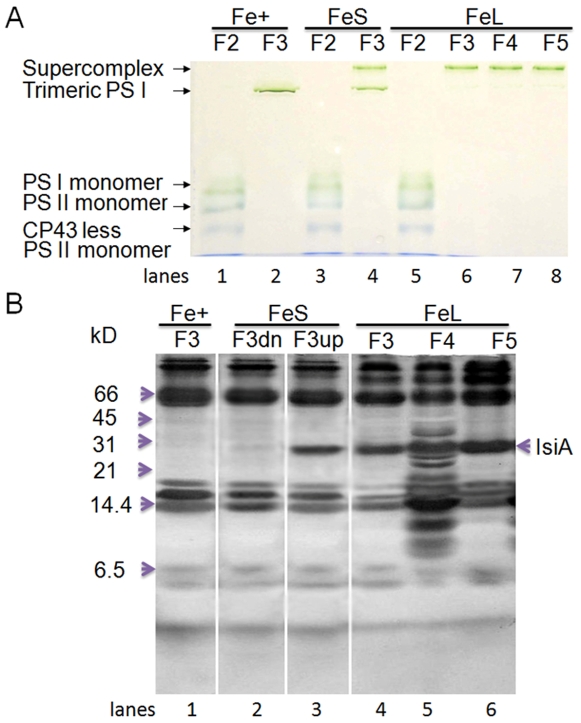
Blue Native and SDS-PAGE analyses of the sucrose-gradient fractions. Sucrose-gradient fractions from Fig. 1 were mixed with 1/10 volume of 5% Serva blue G (100 mM BisTris-HCl, pH 7.0, 0.5 M 6-amino-n-caproic acid, 30% glycerol) and separated by Blue Native PAGE (A) Single bands of the BN-PAGE were excised, denatured with a 1.5 X SDS sample buffer for 30 min at room temperature, and separated by SDS-PAGE (B) F3up and F3dn, the upper and lower green bands of the FeS F3 fraction in A, respectively.

We also analyzed the sucrose gradient fractions using blue-native (BN) PAGE. The results are shown in Fig. 3A. All of the F2 fractions (Fig. 3A, lanes 1, 3 and 5) were similar and contained three bands, which are presumed to be the PSI monomers, the PSII monomers and the CP43-less PSII [41], [42]. The F3 (Fig. 3A, lane 2) from the control sample consisted of a single band, the trimeric PSI, as expected. The F3, F4, and F5 (Fig. 3A, lanes 6 to 8) of long-term iron stressed samples each also contained only one band, with a mobility that was slower than that of the PSI trimers (Fig. 3A, lane 2) on the BN gel, supporting the assumption that they are pigment protein supercomplexes. Interestingly, the short-term iron stressed F3 (Fig. 3A, lane 4) had two bands on the BN gel; one band had a mobility similar to the control F3 band (Fig. 3A, lane 2) and the other was similar to the F3 band of the long-term iron stressed samples (Fig. 3A, lane 6). The protein complexes of the FeL-F3, F4 and F5 (Fig. 3A, lanes 6 to 8) are so large that they barely moved beyond the interface between the stacking and resolving gel, therefore, their relative differences in mobility were not resolved under the conditions we used.

To verify the composition of the pigment protein complexes, the green bands derived from the fractions of F3 (Fig. 3A, lanes 2, 4 and 6), F4 (Fig. 3A, lane 7) and F5 (Fig. 3A, lane 8) were sliced, denatured and separated using 2D SDS-PAGE (Fig. 3B). Clearly, the protein profiles of the lower band of the short-term iron stressed samples (Fig. 3B, lane 2) were the same as those of the control-F3 (Fig. 3B, lane 1) green band, the PSI trimer band. The upper band (lower mobility on the BN gel; Fig. 3B, lane 3) contained an extra protein, the IsiA protein (see Fig. 2 for identification). We assigned this complex as IsiA-PSI trimers. The protein profiles of the F3, F4, and F5 (Fig. 3B, lanes 4 to 6) green bands on the BN gel of the long-term iron stressed samples were similar to those of the corresponding sucrose gradient fractions (Fig. 2A, lanes 6 to 8). We assigned the F3, F4, and F5 of the long-term iron stressed samples as the IsiA-PSI trimer, IsiA-PSI-PSII supercomplex, and IsiA-PSI supercomplex (heavier in density than IsiA-PSI trimer), respectively. Therefore, as iron depletion progressed, the PSI trimers (F3) were converted to the IsiA-PSI supercomplex (F3 of FeL), and in the meantime, the IsiA-PSI-PSII supercomplex (F4) and a heavier IsiA-PSI supercomplex (F5) became apparent (Fig. 1).

### 77K fluorescence of the sucrose-gradient fractions

**Figure 4:**
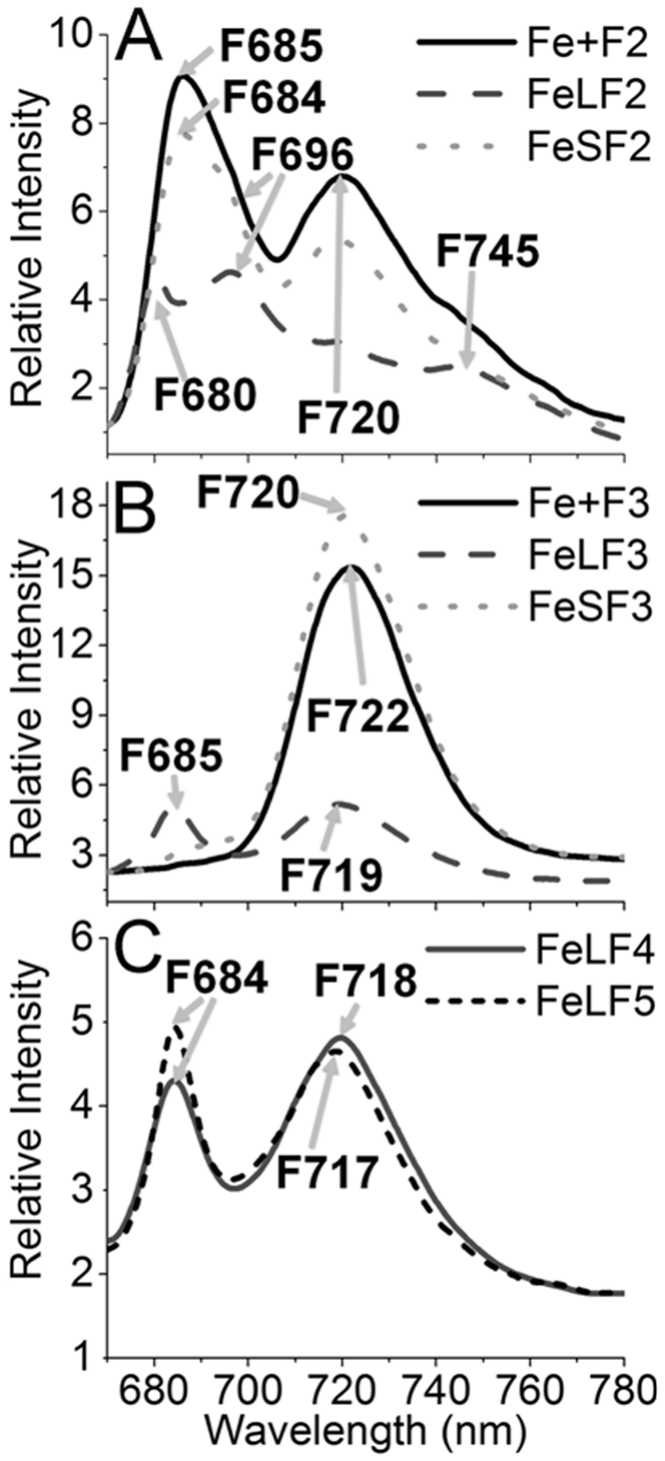
77K fluorescence of the sucrose-gradient fractions. The fluorescence emission spectra of F2 (A), F3 (B), F4 and F5 (C) of the wild type were recorded with excitation at 430 nm in liquid nitrogen. All samples contained 15 µg of Chl/mL.

To further characterize the pigment protein complexes observed, the sucrose-gradient fractions were excited at 430 nm in liquid nitrogen, and the 77k fluorescence emission spectra were recorded. Fig. 4 shows the 77K fluorescence emission spectra of the sucrose gradient fractions. The F2 fraction of wild type cells grown in the LL iron replete media showed a F685 and a F720 peak, which originated from PSII and PSI, respectively [43]. This is in good agreement with the analysis using SDS-PAGE (Fig. 2). The two peaks were reduced when the wild type cells were subjected to short-term iron depletion (Fig. 4A). In addition, the F685 was shifted to F684, concomitant with the appearance of two shoulders, one at F696 and another at F745. Longer-term iron limitation resulted in a further decrease in the overall fluorescence intensity (on a per Chl basis), this was accompanied by a more pronounced blue-shift of F685 to F680 and the appearance of F696 and F745. Therefore, iron starvation strongly affects the spectroscopic properties of the PSI and PSII.

The F3 of the wild type cells (Fig. 4B) exhibited the typical 77K fluorescence spectrum of a PSI trimer with a single peak at 722 nm. After a short-term iron starvation (3 days), the peak position shifted to F720 and two minor peaks, or shoulders, appeared at 686 nm and 695 nm. Under longer-term iron starvation (10 days), the fluorescence peak shifted to 719 nm, and the fluorescence intensity decreased dramatically. A peak at 685 nm became prominent, indicating the formation of the IsiA-PSI supercomplex [26]. This correlates well with the results shown in Fig. 2 and Fig. 3.

The F4 fraction showed two peaks at 684 nm (PSII peak) and 718 nm (PSI peak) (Fig. 4C), that are consistent with the results presented in Fig. 2 and Fig. 3. This further supports that F4 is an IsiA-PSI-PSII supercomplex. The F5 fraction (Fig. 4C) showed one predominant peak at 717 nm (originated from PSI) and a smaller peak at 684 nm (possibly originated from IsiA). This result correlates well with the results of the SDS-PAGE (Fig. 2) and the BN-SDS-PAGE (Fig. 3), supporting our assignment of the F5 as IsiA-PSI supercomplex.

### Impacts of *isiA* deletion on photosynthetic characteristics

**Figure 5:**
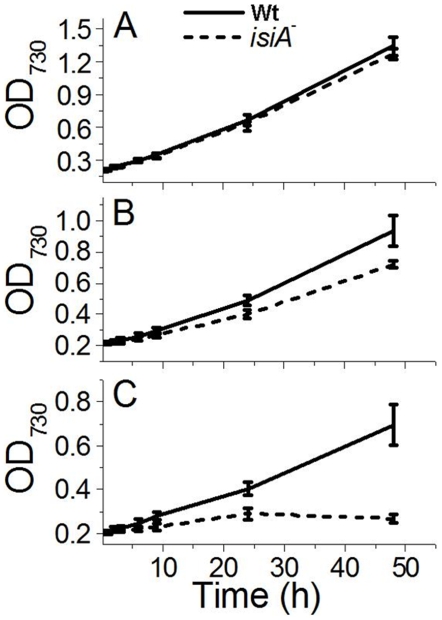
Growth of the *Synechocystis* PCC 6803 strains under different light conditions. Wild type (Wt) and *isiA* ^-^ (*isiA* ^-^) cells in mid-logarithmic growth phase (OD_730_ ∼0.6–0.8) were diluted to an OD_730_ of 0.2 with fresh BG-11 medium and incubated in LL (A), intermediate HL (IHL, B), or HL (C). The growth of the cells was monitored as a change in the optical density at 730 nm. Curves were generated by averaging the data obtained from 6 representative experiments.

It was reported that the deletion of *isiA* from *Synechocystis* resulted in cell death in HL (Havaux et al., 2005). To gain more insights into the mechanisms of this photosensitive phenotype of the *isiA* mutant, we examined the growth and photosynthetic characteristics of the mutant under different light conditions. *Synechocystis* cells in the mid-logarithmic growth phase were diluted with fresh medium to an OD_730_ of 0.2, and then exposed to intermediate HL (IHL) at 200 µmol of photon m^−2^ s^−1^ or to HL at 400 µmol of photon m^−2^ s^−1^ for various lengths of time at 30°C. Cell growth was monitored as a change in optical density. As shown in Fig. 5, the *isiA* ^-^ mutant grew at a similar rate as the wild type in LL (Fig. 5A). Under the IHL conditions, the growth of the *isiA* ^-^ mutant was impeded after 24 h of exposure (Fig. 5B). The *isiA* ^-^ mutant survived the first 24 h in HL, after which the cell growth was completely arrested (Fig. 5C).

**Figure 6:**
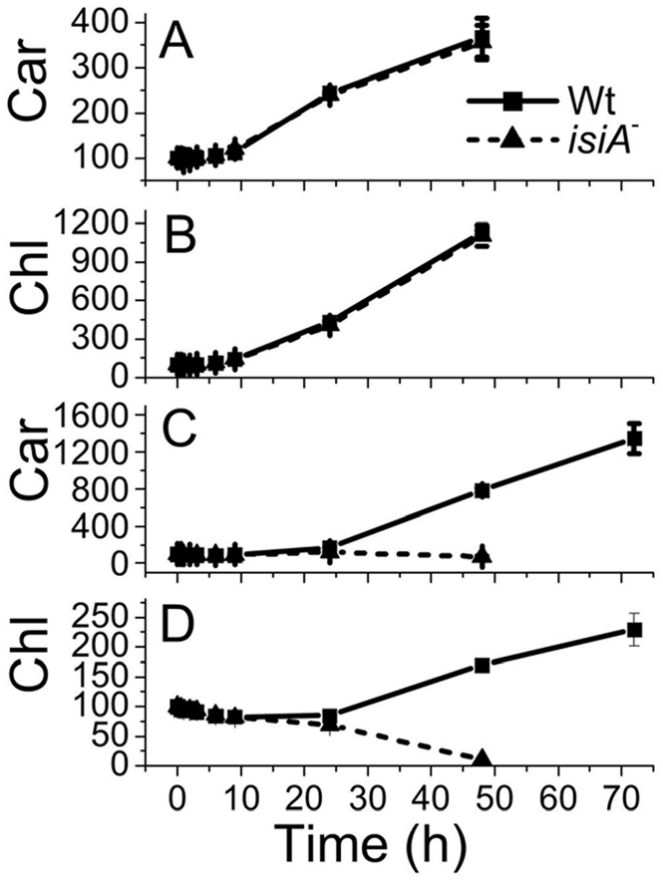
Accumulation of Chl and carotenoids in *Synechocystis* PCC 6803 strains under different light conditions. Wild type (Wt) and *isiA* ^-^ (*isiA* ^-^) cells in the mid-logarithmic growth phase (OD_730_ ∼0.6–0.8) were diluted to an OD_730_ of 0.2 with fresh BG-11 medium and incubated in LL (A, B) or HL (C, D). Samples were taken at different time points for the quantification of carotenoids (A, C) and Chl (B, D). The pigment amount/ml culture at time 0 was normalized to 100% to aid comparison. Curves were generated by averaging the data obtained from six representative experiments.

We also monitored the pigment (Chl and carotenoids) accumulation in the cultures. Both Chl and carotenoids accumulated in the mutant at rates similar to the wild type in LL (Fig. 6A, 6B). The pigments (carotenoids and Chl) of the *isiA* ^-^ mutant started diminishing after 6 h of exposure to HL (Fig. 6C, 6D). The *isiA* ^-^ cells were completely bleached and there were essentially no Chl or carotenoids accumulated in the cells after 48 h of HL exposure. In contrast, the wild type cells adapted well to HL with an enhanced accumulation of carotenoids (Fig. 6). Apparently, the *isiA^-^* mutant had started losing pigment (after 6–12 h in HL) before cell division stopped (after 24 h in HL). Therefore, the failure to accumulate carotenoids might be one of the defects leading to the light sensitivity of the *isiA* ^-^ mutant.

**Figure 7:**
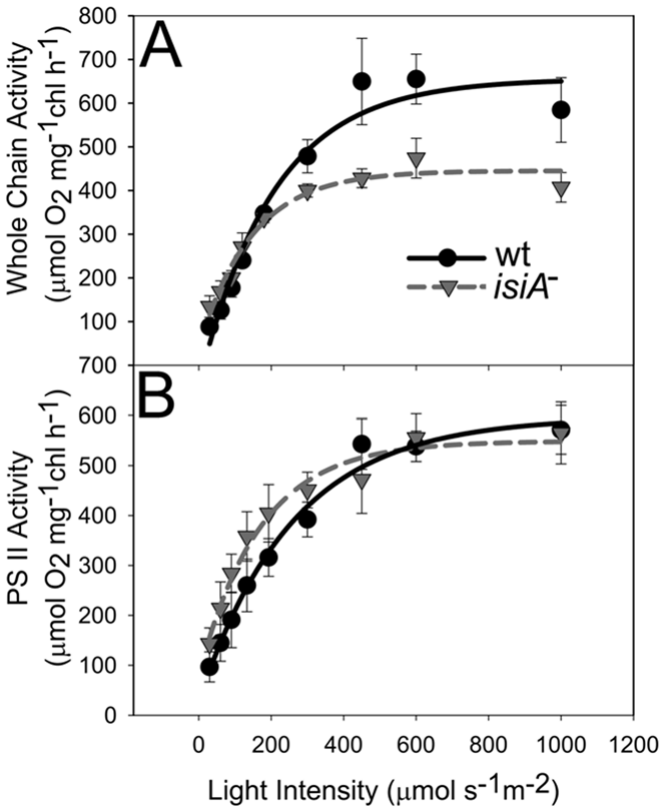
Light response curves of *Synechocystis* PCC 6803 strains. Cultures (OD_730_ approximately 0.6–0.8) were adjusted to a Chl content of 5 µg/ml with fresh BG-11 medium. The activities of the whole-chain (A) and PSII-mediated (B) electron transport were evaluated by measuring the oxygen evolution at various light intensities as described in the Material and Methods section. Curves were generated by averaging the data obtained from 5 representative experiments.

To study the influence of *isiA* deletion on the light usage of both the whole-chain and the PSII- mediated electron transport activities, we cultured the wild type and *isiA^-^* strains in BG-11 under LL conditions. We then monitored the oxygen evolution activities of the cells under different light conditions in the presence or absence of artificial electron acceptors using a Clark-type electrode. Fig. 7A presents the light-response curve of the whole-chain electron transport. Apparently, the *isiA* ^-^ strain can utilize low intensity light as efficiently as the wild type because the initial slopes of the light response curves (the apparent quantum yield or the AQY) are similar for the two strains (Table 1). As the light intensity increases, the whole-chain electron transport of the mutant lags. The light response curve of the *isiA* ^-^ mutant reached an earlier and lower plateau (the saturated carbon assimilation rate or the Asat) than that of the wild type strain (Table 1). This correlates well with the growth characteristics of the *isiA^-^* mutant: it grows at a rate similar to the wild type in LL, but at a reduced rate in IHL (Fig. 5B).

**Table 1:** The apparent quantum yield (AQY) and the saturated carbon assimilation rate (Asat) of PSII and the whole-chain electron transport.

	Whole Chain		PSII	
	AQY	Asat	AQY	Asat
Wt	1.76±0.24	656±101**	1.58±0.22	587±49
*isiA-*	1.65±0.16	445±70	1.71±0.21	549±63

N = 5,

**, P<0.05.

No significant differences were observed in either the Asat or AQY between the wild type and the *isiA^-^* mutant (Fig. 7B, Table 1) when the PSII-mediated electron transport was measured in the presence of an artificial electron acceptor. Thus, the PSII is fully functional in the *isiA^-^* mutant.

### Levels of D1 protein and lipid peroxidation

**Figure 8:**
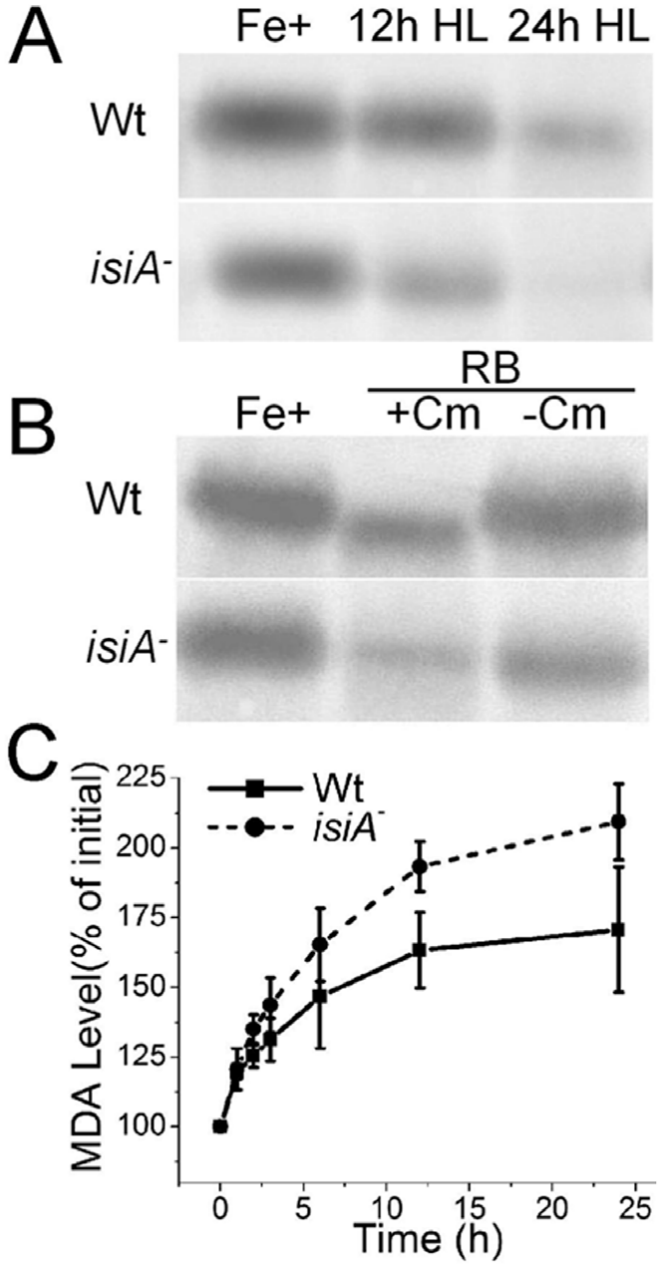
Sensitivity of the *isiA* ^-^ mutant to oxidative stress. A and B, D1 protein level in HL (A) or RB (B) treated cells in the presence (+Cm) or absence (−Cm) of chloramphenicol. Wild type (Wt) and *isiA* ^-^ (*isiA* ^-^) cells in mid-logarithmic growth phase (OD_730_ ∼0.6–0.8) were diluted to an OD_730_ of 0.2 before exposure to HL. Blots of the thylakoid membrane proteins were probed with polyclonal anti-D1 antibodies. C, The level of lipid peroxidation. The cells were exposed to HL for various length of time as indicated. Malondialdehyde (MDA) level was measured using the BIOXYTECH lpo-586 kit. Curves were generated by averaging the data obtained from 3 representative experiments.

The sensitivities of the wild type and the *isiA^-^* strains to treatments with oxidants and HL were assessed by Western blot analysis (Fig. 8A, 8B). The D1 protein levels of the *isiA^-^* mutants were at about 50% of the wild type levels when the cells were treated with HL for 12 h, and were ∼20% of the wild type levels when the HL treatment was extended to 24 h (Fig. 8A). The D1 protein level of the *isiA^-^* mutant was also ∼50% of the wild type when treated with Rose Bengal for 2 h (Fig. 8B). In the presence of chloramphenicol (200 µg ml^−1^), a protein synthesis inhibitor [44], [45], the level of the D1 protein in the *isiA* ^-^ mutant dropped to less than 30% of the wild type level. These results indicated that the *isiA^-^* cells are more sensitive to oxidative stress than the wild type.

The lipid peroxidation, a commonly accepted indicator of oxidative stress, was assayed by measuring the contents of malondialdehyde (MDA) in cells treated by HL. The results are shown in Fig. 8C. The MDA levels in the mutant strain were about 50% higher than those of the wild type cells at any given point when the cells were subjected to HL. Clearly, HL treatment resulted in extensive membrane damage to the *isiA^-^* cells.

### State transition and blue light-induced quenching

**Figure 9:**
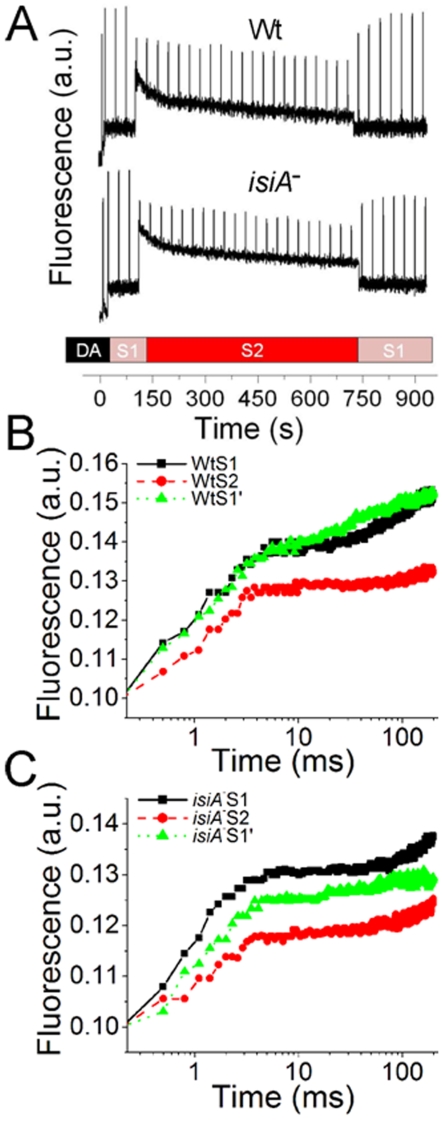
State transition of *Synechocystis* PCC 6803 strains. A, Typical traces of the state transition analysis. Wild type (Wt) and *isiA* ^-^ (*isiA* ^-^) cells in mid-logarithmic growth phase (OD_730_ ∼0.6–0.8) were collected and resuspended to an OD_730_ of 0.6 with fresh BG-11 medium. State transition was analyzed by Chl fluorescence. A far-red light was first applied to the dark-adapted cyanobacterial cultures to fully oxidize PSI, which was followed by a series of saturation flashes to determine the S1 level. An actinic light of 100 µ mol photon m^−2^ s^−1^ was subsequently turned on to induce photosynthesis. The S2 level was then assessed by a saturation pulse before the far-red light was applied again to drive the S2-S1 transition. B and C, Fast fluorescence induction kinetics of the wild type (B) and *isiA* ^-^ (C) strains at state 1 (S1), state 2 (S2), and state transition level (S1′).

State transition is a physiological adaptation mechanism that balances the distribution of the light energy absorbed by phycobilisomes between PS I and PS II in cyanobacteria [46], [47]. The state transition capability of the *isiA^-^* mutant was analyzed using Chl fluorescence analysis. A far-red light was first applied to the dark-adapted cyanobacterial cultures to fully oxidize PSI, which was followed by a series of saturation flashes to determine the S1 level. An actinic light of 100 µ mol photon m^−2^ s^−1^ was subsequently turned on to induce photosynthesis. The S2 level was then assessed by a saturation pulse before the far-red light was applied again to drive the S2-S1 transition. The typical traces of state transition measurements of the wild type and the *isiA* ^-^ mutant are shown in Fig. 9A. The wild type culture had an approximately 100% state transition, as indicated by the fast and full relaxation of Fm. Therefore, the photoinhibitory effect of such light intensity to the wild type is negligible. In contrast, the *isiA* ^-^ mutant exhibited a very low state-transition level and a substantial photoinhibitory quenching, as indicated by its poorly recovered Fm. To take a closer look at the state-transition defect of the *isiA^-^* mutant, the fast kinetics of the fluorescence induction of both the wild type and the *isiA^-^* mutant at different states were examined and plotted, as shown in Fig. 9B and 9C. The plateau of the induction curves represents the Fm, i.e., the maximum level of fluorescence. It is apparent that the wild type had a fully relaxed Fm (state transition) (Fig. 9B). In contrast, the Fm of the *isiA^-^* mutant recovered only ∼34% (Fig. 9C), and retained a large portion of non-relaxable photoinhibitory quenching. The defect in state transition of the *isiA* ^-^ mutant was also observed by fluorescence emission spectroscopy at 77K (data not shown), however, this traditional method showed a rather large standard error between observations.

We also performed blue light induced quenching analysis with a 10 nm bandpass filter at 485 nm (which includes the carotenoid absorption but excludes the Chl absorption). No differences were observed between the wild type and the mutant (data not shown). Apparently the deletion of *isiA* does not affect the blue light-induced and the orange carotenoid protein (OCP)-related quenching processes under HL conditions, which is in agreement with previous reports [38], [39]. These finding might appear to be inconsistent with two other reports [36], [37]; however, these authors performed their studies under iron limitation conditions rather than HL conditions.

## Discussion

The *isiA* gene is one of the most dynamic genes in cyanobacteria. The regulation of the gene occurs at both transcriptional and translational levels and involves at least two regulators, the Fur protein and the *isrR* microRNA [48]. Although *isiA* transcripts accumulate under various growth conditions, the IsiA proteins were detected only under the iron limitation [18], [24], [49] and recently under high light conditions [25]. It is known that 18 subunits of IsiA encircle the trimeric PSI to form a supercomplex under iron starvation conditions [26], [27]. The IsiA protein can also form aggregates around the PSI monomer or in the total absence of PSI under longer-term or extensive iron starvation conditions [30], [31]. The functions of IsiA in these complexes were not well established, and many functional propositions were deduced exclusively from the physiological analysis. The IsiA-PSI trimer supercomplex might dissipate the excess light energy [32], [33]. It might also be involved in the blue light-induced reversible non-photochemical fluorescence quenching under iron starvation conditions [36], [37]. Regardless of the ambiguity in functions, IsiA is important for the survival of cyanobacterial cells under HL conditions as reported [18]. This is confirmed by the study reported here as well (Fig. 5). It is also clear that the association of IsiA with PSI causes a 2-nm blue shift of PSI fluorescence at 77K (from 722 nm to 720 nm) (Fig. 4, and also see [11], [12]. The decrease of PSI fluorescence at 720 nm (Fig. 4) indicates that the PSI is in a strongly quenched state, which is in agreement with previous reports [32], [50]. The iron starved wild type cells also showed a decreased F720 (F717)/F685 ratio, which may reflect the presence of PSI-less IsiA aggregates [31] since the IsiA-PSI supercomplexes have a very small 685 nm emission [28], [31].

An important finding made in this article is that different IsiA-PSI supercomplexes were observed in cells that underwent extensive iron depletion (Fig. 1). In addition to the IsiA-PSI supercomplexes (F5 and the PSI trimer fraction F3), we observed a fraction (F4) with a density higher than that of the PSI trimer fraction (F3). This fraction contained IsiA and the PSI and PSII proteins (F4) (Fig. 2 and Fig. 3). Because the fraction had a density higher than that of the PSI trimers (Fig. 1), exhibited typical PSI and PSII fluorescence peaks at 77K (Fig. 4C), ran as a single green band on BN gel (Fig. 3A), and show typical profiles as mixture of PSI and PSII when resolved by SDS-PAGE (Fig. 2A, Fig. 3B), it is likely that this fraction consisted of a novel PSI-PSII supercomplex. The IsiA proteins in F2 are likely to be IsiA aggregates, because the association of PSI or PSII with IsiA will increase the density of the protein complexes as in the case of the IsiA-PSI trimer complexes. The 77K fluorescence emission of F3 revealed that the PSI trimers were in a strongly quenched state (Fig. 4B). This indicates a role played by the IsiA-PSI supercomplex in non-photochemical quenching, as observed earlier by Ihalainen et al. [32]. The IsiA-PSI and the IsiA-PSI-PSII supercomplexes were also observed in cells subjected to HL treatment. The quantity of these complexes was fairly low in HL as compared with the iron limitation conditions, which correlates well with the lower amount of IsiA protein present in the HL grown cells. However, the amount of the IsiA or IsiA-containing PSI complexes may be not critical for proper function, since the deletion of IsiA resulted in the diminishment of the trimeric PSI complexes, even under LL and iron replete conditions (Fig. 1), when the IsiA protein level was very low. Therefore, the role of IsiA in the formation/integrity of these higher complexes is more than physically encircling the complexes, but rather playing a more active role (e.g., regulator or assembler).

The physiological significance of the IsiA-PSI-PSII supercomplexes (F4) remains to be determined. Some insights may come from the results that demonstrated the state transition defect in the *isiA* ^-^ mutants (Fig. 9). It is tempting for us to hypothesize that within the IsiA-PSI-PSII complexes, the PSII and PSI can interact more directly, favoring or enhancing the physiological processes that require direct interaction between PSI and PSII, such as state transition. This would help protect PSII and/or reduce the production of reactive oxygen species under stress conditions (Fig. 8). The *isiA* ^-^ mutant lacked F3, F4 and F5 (Fig. 1) under either iron-limitation or HL conditions and suffered from extensive oxidative stress (Fig. 5, Fig. 8). We thus propose that the IsiA protein, induced by various stresses, promotes the formation of (1) the IsiA- trimeric PSI supercomplex, favoring non-photochemical quenching through PSI and (2) the IsiA-PSI-PSII supercomplex, favoring processes involving both photosystems such as state transition. Under relatively low light and iron-sufficient conditions, the IsiA-PSI-PSII supercomplex might also play an important role, as a low amount of the supercomplex was also detected (data not shown). It is possible that the two photosystems might interact with each other transiently in LL. The supercomplex may also play other active, non-structural roles in stress defenses. These processes help to alleviate oxidative damages, and protect cyanobacterial cells from excess excitation.

## Materials and Methods

### Growth conditions and treatments

*Synechocystis* cells were cultivated in a BG-11 medium with 10 mM TES, at pH 8.2 and 30°C. The culture was bubbled with air under low light (LL) conditions (40 µmol m^−2^ s^−1^), intermediate high light (IHL) conditions (200 µmol m^−2^ s^−1^), or high light (HL) conditions (400 µmol m^−2^ s^−1^).

For the IHL and the HL experiments, the cells reaching a mid-logarithmic growth phase (OD_730_ approximately 0.6 to 0.8) were diluted with fresh medium to OD_730_ = 0.2 and exposed to IHL or HL for various durations of time.

Iron free BG11 was acquired by method described by [40]. For iron starvation, the mid-logarithmic growth phase (OD_730_ approximately 0.6 to 0.8) cultures were pelleted at room temperature, resuspended and washed sequentially with 10 mM TES (pH 8.2), and iron-free BG11. The cultures were then resuspended with iron-less BG-11 to OD_730_ = 0.2 and grown for either 3 (short term) or 10 (long term) days.

Mid-logarithmic growth phase (OD_730_ approximately 0.6 to 0.8) cultures were diluted with fresh medium to OD_730_ = 0.2, artificial oxidant Rose Bangal (RB) was applied at a concentration of 10 µM for 2 h under low light growth condition.

### Estimation of Chl a and carotenoid concentration

The Chl *a* and carotenoids concentration was estimated from the dimethylfluoride extract using the formula developed by Moran [51].
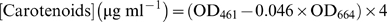
(2)

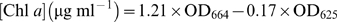
(1)


### Thylakoid membrane preparation and fractionation of membrane protein complexes

Thylakoid membranes were prepared as previously described [25] with some modifications. Briefly, the cell pellets derived from cells grown to the mid-logarithmic phase were resuspended in ice-cold thylakoid buffer (50 mM 3-(N-morpholino)-propanesulfonic acid, pH 7.0, 0.4 M sucrose, 5 mM MgCl_2_, 5 mM CaCl_2_, 1 mM freshly made phenylmethylsulfonyl fluoride). The cells were broken in a Bead-Beater with an ice-jacketed sample chamber by six breakage cycles at full speed (30 s of bursts, followed by 5 min of chilling) after adding an equal volume of glass beads pre-wetted by the thylakoid buffer to the cell suspension. The homogenate was centrifuged at 1,800×*g* for 10 min to remove the unbroken cells, the cellular debris and the glass beads. The membranes in the supernatant were then spun down at 50,000×*g* at 4°C for 60 min. After washing with 2 mM dodecyl maltoside to remove any of the remaining phycobilisomes, the membranes were washed twice and resuspended in the thylakoid buffer to a Chl concentration of 1 mg ml^−1^.

To fractionate the membrane protein complexes, a solution of 10% (the percentage (%) of a solution is calculated as weight to volume (w/v) unless otherwise indicated) dodecyl maltoside was added to the thylakoid membranes to achieve a detergent-to-Chl ratio of 15∶1. The membrane was solubilized at 4°C for 30 min before it was loaded onto a 10–30% (w/w) step sucrose gradient and it was then centrifuged at 160,000×*g* for 16 h at 4°C. The pigmented fractions were collected and stored at −80°C until use.

### Blue native, tricine SDS PAGE, and Western blot analysis

Blue native PAGE was performed as described [25]. Different sucrose gradient fractions were collected and combined with 1∶10 volume of 10X sample buffer (5% Serva G; 25 mM BisTris-HCl, pH 7.0; 250 mM 6-amino-caproic acid; 10 mM EDTA; 30% sucrose). The prepared samples were then loaded onto the gel, and run at a constant voltage of 100 V.

For denatured electrophoresis, a 1/3 volume of the 4X SSB (SDS sample buffer) buffer (200 mM Tris-HCl, pH 6.8, 8% SDS, 400 mM DTT, 0.02% bromophenol blue) was added to the sample, and incubated at 75°C for 10 min. The denatured sample was then loaded onto a 12–20% tricine-SDS gel with 6 M urea as described [52].

For the western blot analysis, the SDS-gel was blotted onto a nitrocellulose membrane, incubated with specific primary antibody, and subsequently incubated with the HRP-linked secondary antibody. The hybridized proteins were then detected by chemiluminescence.

### Electron transport activity

The whole-chain or PSII-mediated electron transport rates were estimated by measuring the O_2_ evolution using a Clark-type electrode. Cultures were adjusted to a Chl content of 5 µg ml^−1^ with fresh BG-11 for all of the measurements. The whole-chain electron transport (H_2_O to CO_2_) rate was measured using water as the electron donor in the presence of 1 mM NaHCO_3_. The PSII-mediated reaction mixture contained 5 mM NH_4_Cl,4 mM K_3_FeCN, 1 mM phenyl-p-benzoquinone, and 40 mM Tricine (pH 7.5), which was used to measure the electron transport rate from H_2_O to phenyl-p-benzoquinone via PSII. The O_2_ evolution was followed at various light intensities for 3 min and the rate was calculated accordingly.

### Chl fluorescence analysis and 77K fluorescence spectroscopy

The Chl fluorescence analysis was performed using a Dual-PAM-100 P700 & Chl Fluorescence Measuring System (Heinz Walz, Germany) as described in Wang et al. [25]. Fluorescence emission spectra at 77K were recorded with excitation and emission bandwidths of 5 nm and 1.5 nm, respectively, using a Cary Eclipse fluorescence spectrophotometer (Varian Inc., Palo Alto, CA). The excitation wavelength used was 430 nm. The thylakoid membranes were adjusted to a Chl concentration of 15 µg ml^−1^.

### Lipid peroxidation assessment

Lipid peroxidation was assessed by measuring the amount of malondialdehyde (MDA), which is the decomposed product of polyunsaturated fatty acid peroxides. The MDA was quantified using the BIOXYTECH® LPO-586™ kit (OXIS International Inc.) according to the manufacturer's instructions. This assay is based on the reaction of a chromogenic reagent *N*-methyl-2-phenylindole with the MDA at 45°C to yield a stable chromophore with a maximal absorbance at 586 nm.

### DNA manipulation and mutant construction

**Figure 10:**
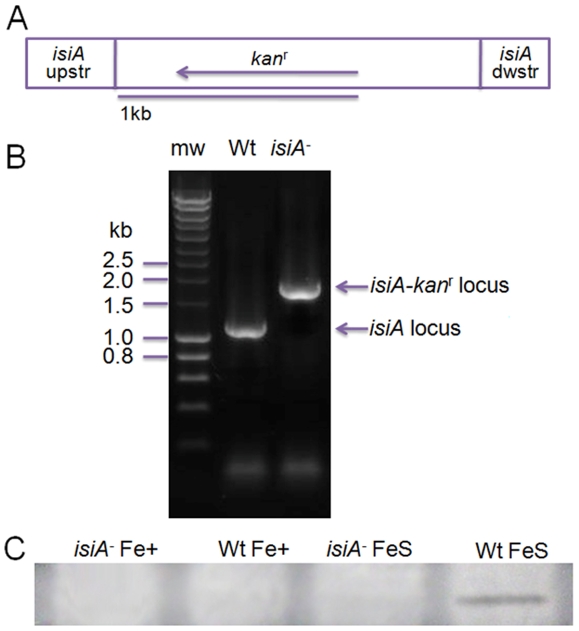
Inactivation of the *isiA* gene. A, Depiction of the plasmid construct used to generate the *isiA* ^-^ mutant. The plasmid carries a 135-bp deletion of *isiA* coding sequence and an insertion of the kanamycin resistant cartridge (*kan* ^r^). B, PCR analysis of the *isiA* gene. Genomic DNA for PCR analysis was isolated from the wild-type (Wt) and a putative *isiA* ^-^ mutant (*isiA* ^-^). C, Western blot analysis of IsiA. Cells were grown in LL in normal (Control) or iron-free (FeS) BG 11 medium. Blots of the thylakoid membrane proteins were probed with polyclonal anti-IsiA antibodies. mw, DNA size marker.

To inactivate the *isiA*, we amplified a 1661-bp DNA fragment (corresponding to 375-bp upstream of the *isiA* start codon to 260-bp downstream of the stop codon) from *Synechocystis* PCC 6803 genomic DNA. The primers used were 5′-ATAAGATCTTTTACACCGCTACTGC-3′ and 5′-GACACTGACAGCAATACCAAACTAA-3′. The DNA fragment covering the entire *isiA* gene was cloned into a pGEM -T Easy Vector (Promega). A 135-bp coding sequence of *isiA* was then deleted by digestion with restriction enzymes (*Bsa*BI and *Pml*I) and replaced with a 795-bp kanamycin resistance cartridge. The resulting plasmid, pGEM-T-*isiA*-kan^r^, was used to transform the *Synechocystis* PCC 6803 wild type cells. The transformants were selected by screening for resistance to 10 µg ml^−1^ kanamycin in the BG-11 medium. Transformants were restreaked onto the same medium, and the segregation of the inactivated *isiA* gene was monitored by PCR, using genomic DNA from the transformants. The two primers used were 5′-CTGATCAGTCTGGGCTTTTTATTG-3′ and 5′- CTAGGTTTGCAAGGAATCAAAC -3′ to recognize sequences around the start and stop codons of the *isiA* gene, respectively. The expected fragment sizes of the wild type *isiA* and the interrupted *isiA* loci are 1029 bp and 1689 kb, respectively. Homoplasmic mutants were obtained and evaluated by PCR (Fig. 10B), which was further verified by a western blot analysis of IsiA (Fig. 10C). The mutant generated was designated as *isiA^-^*.
